# FOXC1 plays a crucial role in the growth of pancreatic cancer

**DOI:** 10.1038/s41389-018-0061-7

**Published:** 2018-07-06

**Authors:** Ramadevi Subramani, Fernando A. Camacho, Carly Ivy Levin, Kristina Flores, Alexa Clift, Adriana Galvez, Mauricio Terres, Servando Rivera, Sai Navana Kolli, Joshua Dodderer, Megan Miranda, Alejandro Rodriguez, Diego A. Pedroza, Animesh Chatterjee, Rajkumar Lakshmanaswamy

**Affiliations:** 1 Center of Emphasis in Cancer Research, Department of Biomedical Sciences, Paul L. Foster School of Medicine, Texas Tech University Health Sciences Center El Paso, El Paso, Texas 79905 USA; 2 Graduate School of Biomedical Sciences, Texas Tech University Health Sciences Center El Paso, El Paso, Texas 79905 USA; 3 The University of Texas at El Paso, El Paso, TX 79968 USA

## Abstract

IGF-1R signaling controls various vital cellular functions and this signaling is deregulated in many cancers, including pancreatic cancer. Several efforts have mainly focused on inhibiting the IGF-1R signaling cascade. The outcomes of these focused preclinical studies have been positive, whereas clinical trials of IGF-1R inhibitors in pancreatic cancer have failed, raising the questions about this therapeutic approach. This necessitates a better understanding of the role of IGF-1R signaling in pancreatic cancer. We investigated the impact of IGF-1R signaling on crucial transcription factors and identified the FOXC1 as one of the crucial regulator of IGF-1R signaling. We employed genetic approaches to overexpress and silence FOXC1 in pancreatic cancer cells. Our results demonstrate that IGF-1R and FOXC1 seem to positively regulate each other. Further, FOXC1 increased the metastatic abilities of pancreatic cancer cells by enhancing cell proliferation, migration, invasion, epithelial-to-mesenchymal transition, and angiogenesis. The data from xenograft experiments further established the importance of FOXC1 in pancreatic tumorigenesis. In conclusion, FOXC1 is a potent oncogenic transcription factor, which promotes pancreatic cancer growth and metastasis. Thus, targeting FOXC1 could be a potential therapeutic strategy against pancreatic cancer.

## Introduction

Advancement in cancer research has resulted in decreasing mortality associated with various cancers, but mortality associated with pancreatic ductal adenocarcinoma (PDAC) is still very high. PDAC is a highly metastatic and lethal disease with a 5-year survival rate of 7%^1^ and it’s been predicted to become the second leading cause of cancer-related death by 2020^2^. Mono or combined chemotherapy has been the main treatment modality available for PDAC patients. These therapies are not effective; hence, the patients predictably develop recurrent disease^3,4^. Thus, it is essential to develop novel therapeutic strategies against this lethal disease.

Growth factors and their receptors are key mediators that facilitate interactions between tumor cells and the microenvironment^5^. These interactions triggers processes essential for tumor progression, angiogenesis, inflammation, and metastasis^6–10^. Insulin-like growth factor 1 receptor (IGF-1R) is one of the key growth factor receptors, which influences proliferation, metastasis, and radio- and chemo-resistance of various malignancies^11–14^. Recently, we reported that IGF-1R is aberrantly overexpressed in pancreatic cancer cells favoring tumor cell survival, epithelial-to-mesenchymal transition (EMT), and metastatic pathways^15^. However, the mechanisms underlying these tumor-promoting effects of IGF-1R is still not well understood.

FOXC1 is a member of the forkhead box family of transcription factors (TFs) and has been shown to have a critical role in embryogenesis, differentiation, and angiogenesis^16,17^. FOXC1 is highly expressed in breast and liver cancers^18,19^, and is linked to poor prognosis due to enhanced EMT and drug resistance through several intermediaries such as nuclear factor-κB^20^. Recent studies suggest that FOXC1 has a direct correlation with angiogenic factor vascular endothelial growth factor (VEGF)-A^21^. Other studies have demonstrated that FOXC1 regulates cancer stem cell properties through the activation of smoothened-independent Hedgehog signaling^17^. Our preliminary studies demonstrated that the activity of FOXC1 was highly downregulated in IGF-1R-silenced PDAC cell lines. In the current study, for the first time we show that FOXC1 mediates the pro-tumorigenic effects of IGF-1R. Our results demonstrate an important role for IGF-1R–FOXC1 signaling axis in pancreatic tumorigenesis.

## Results

### Crosstalk between IGF-1R and FOXC1

**Fig. 1:**
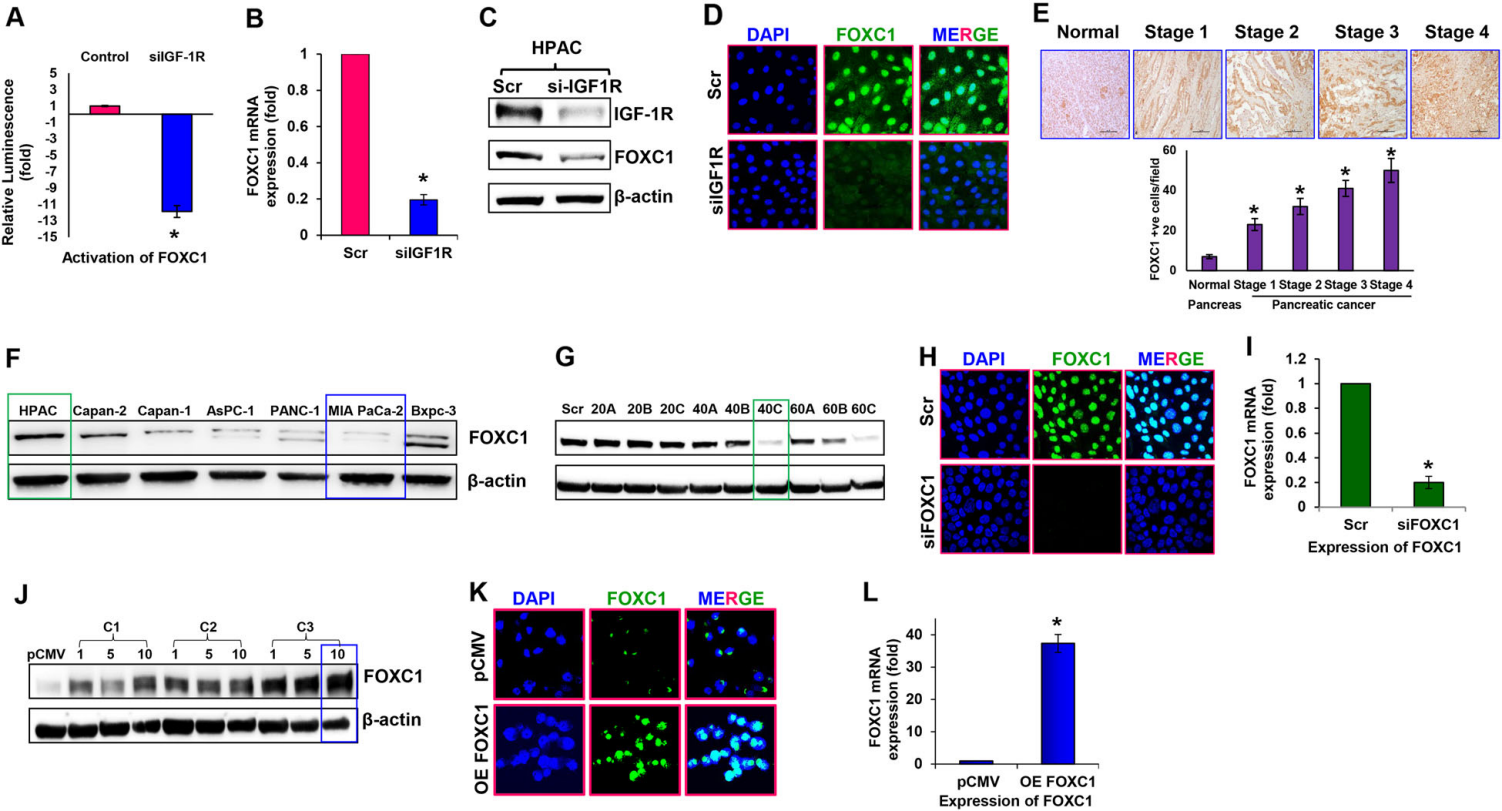
Silencing and overexpression of FOXC1 in pancreatic cancer cells. **a** Activity of FOXC1 transcription factor in IGF-1R-silenced HPAC cells. **b** Relative mRNA expression level of FOXC1 was assessed by real-time RT-PCR in IGF-1R-silenced HPAC cells. **c** Western blot analysis of IGF-1R and FOXC1 expression in IGF-1R-silenced HPAC cells. **d** Representative photographs of immunofluorescence analysis of FOXC1 expression in IGF-1R-silenced HPAC cells were visualized via confocal microscopy at × 60 magnification. **e** Representative qualitative (FOXC1-positive cells indicated by brown staining) and quantitative immunohistochemical analysis (bar diagram represents # of FOXC1-positive cells/field) of FOXC1 by tumor stage in human pancreatic ductal adenocarcinoma tissues and in normal pancreas tissue at × 40 magnification. IHC staining was quantitated at five random fields per section with at least 200 cells/field. **f** Western blot analysis of FOXC1 expression in a panel pancreatic cancer cell lines (HPAC, Capan-2, Capan-1, AsPC-1, PANC-1, MIA PaCa-2, and BxPC-3). **g** HPAC cells were transfected with three predesigned FOXC1 siRNAs (labeled as A, B, and C) at concentrations of 20, 40, and 60 nM along with scrambled control siRNA (Scr). Silencing efficacy of FOXC1 siRNA was determined using western blot analysis. **h** Representative photographs of immunofluorescence analysis of FOXC1 expression in FOXC1-silenced HPAC cells at × 60 magnification. **i** Relative mRNA expression level of FOXC1 was assessed by real-time PCR in FOXC1-silenced HPAC cells. **j** MIA PaCa-2 cells were transfected with FOXC1-overexpressing plasmid DNA from three different colonies (labeled as C1, C2, and C3) at concentrations of 1, 5, and 10 µg along with pCMV control plasmid DNA. Overexpression efficacy of FOXC1 plasmid DNA in MIA PaCa-2 cells was determined using western blotting. **k** Representative photographs of immunofluorescence analysis of FOXC1 expression in FOXC1-overexpressing MIA PaCa-2 cells at × 60 magnification. **l** Relative mRNA expression level of FOXC1 was assessed by real-time PCR in FOXC1-overexpressing MIA PaCa-2 cells. Data shown as mean ± SEM. Experiments (*n* = 3) were repeated three times in triplicates. *p < 0.05

Earlier, we had demonstrated that IGF-1R was upregulated in PDACs and silencing of IGF-1R diminished their proliferative and metastatic capabilities^15^. To understand the influence of IGF-1R on TFs in pancreatic cancer, we used the Transcription Factor Activation Profiling Array and examined the activation levels of various TFs in IGF-1R-silenced HPAC cells. This cell line was chosen among a panel of three pancreatic cancer cell lines screened, because it had the highest expression of IGF-1R^15^. On analyzing the activation levels of 96 different TFs, the data revealed that silencing IGF-1R resulted in altering the activities of various TFs. Among these, FOXC1 was the most significantly downregulated in IGF-1R-silenced HPAC cells (Fig. 1a).

In order to understand the crosstalk between IGF-1R and FOXC1, we assessed the expression of FOXC1 at transcript and protein levels in response to IGF-1R silencing. Our data demonstrated that silencing IGF-1R decreased the expression of FOXC1 at transcript level by ~5-fold (Fig. 1b). Protein expression data further confirmed the reduced levels of FOXC1 in IGF-1R-silenced HPAC cells (Fig. 1c). In addition, immunofluorescence analysis demonstrated that the nuclear expression of FOXC1 was drastically reduced in the IGF-1R-silenced HPAC cells (Fig. 1d). Immunohistochemical analyses of the pancreatic cancer tissue microarray confirmed a higher expression of FOXC1 in PDACs compared with normal pancreas. Furthermore, based on intensity and quantity of the immunostaining, there was a positive correlation between the stage of pancreatic cancer progression and FOXC1 expression (Fig. 1e). Earlier we also observed an increase in expression of IGF-1R in PDACs^15^, similar to the FOXC1 expression observed in the current study further indicating the relationship between IGF-1R and FOXC1.

Further using a panel of seven pancreatic cancer cell lines (HPAC, Capan-2, Capan-1, AsPC-1, PANC-1, MIA PaCa-2, and BxPC-3) we analyzed the expression levels of FOXC1. Immunoblot data showed that there was a range of expression of FOXC1 among the pancreatic cancer cell lines with HPAC having high expression and MIA PaCa-2 having low expression (Fig. 1f). These findings were validated by reverse transcriptase real-time PCR (RT-PCR) analysis, RRN18S was used as the internal control for gene expression analysis (Supplementary Fig. S1).

To understand the role of FOXC1 on pancreatic cancer, it was silenced in HPAC cells and overexpressed in MIA PaCa-2 cells. Three different small interfering RNAs (siRNAs) (subtypes A, B, C) targeting FOXC1 (20, 40, 60 nM) were used to examine the efficacy of silencing FOXC1. Immunoblot analysis showed the effective silencing of FOXC1 in HPAC cells (Fig. 1g). Immunofluorescence and RT-PCR analysis further confirmed the silencing efficacy of FOXC1 siRNA (Fig. 1h, i). To further understand the role of FOXC1 in pancreatic cancer growth, it was overexpressed in MIA PaCa-2 cells (Fig. 1j). The colony that was transfected with 10 μg of FOXC1 pcDNA exhibited the highest expression of FOXC1. These findings were further corroborated by performing immunofluorescence and RT-PCR analysis (Fig. 1k, l). These cell lines (FOXC1-silenced HPAC cells and FOXC1-overexpressing MIA PaCa-2 cells) were used for all our further experiments.

**Fig. 2:**
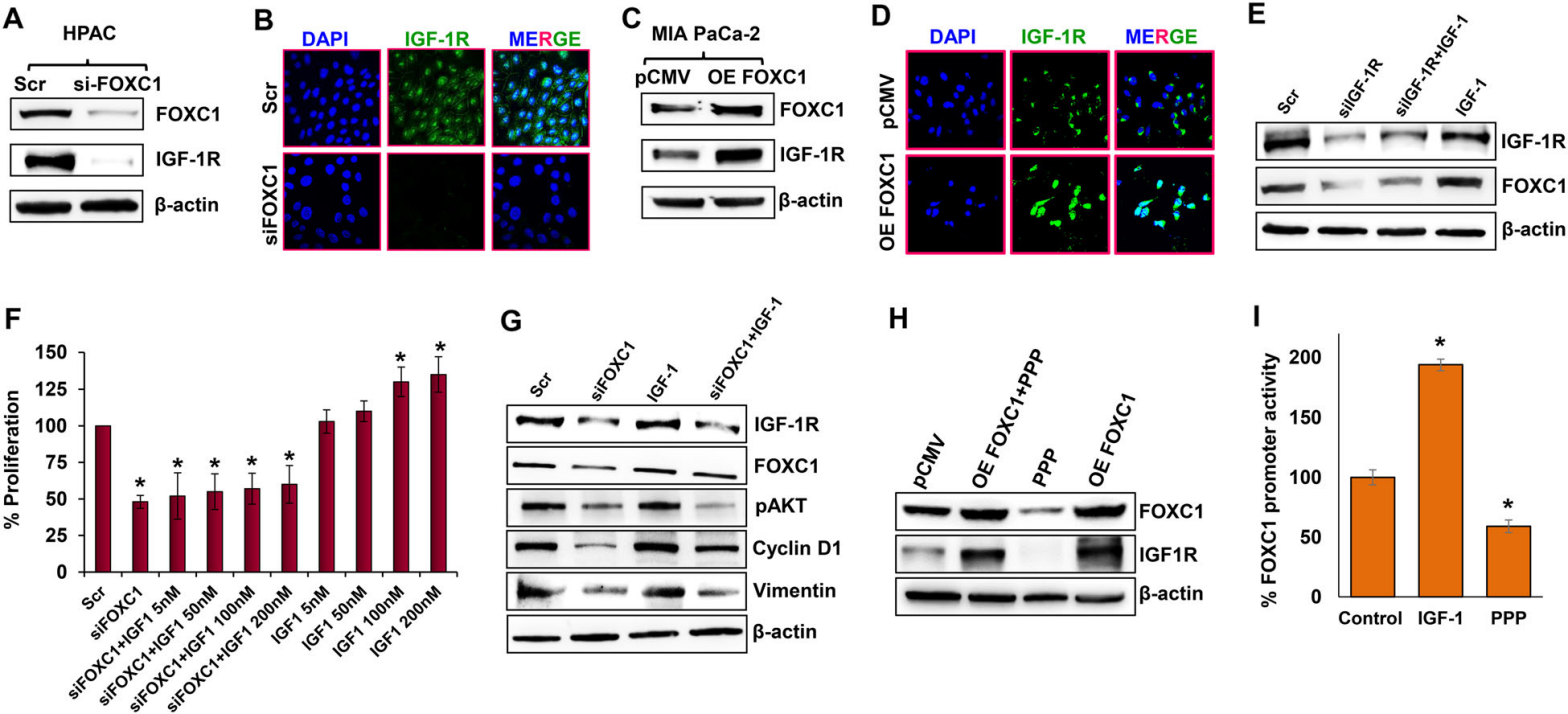
Crosstalk between FOXC1 and IGF-1R in pancreatic cancer cells. **a** Western blot analysis of FOXC1 and IGF-1R expression in FOXC1-silenced HPAC cells. **b** Immunofluorescence analysis of IGF-1R expression in FOXC1-silenced HPAC cells visualized through confocal microscopy at × 60 magnification. **c** Western blot analysis of FOXC1 and IGF-1R expression in FOXC1-overexpressing MIA PaCa-2 cells. **d** Immunofluorescence analysis showed increased IGF-1R expression in FOXC1-overexpressing MIA PaCa-2 cells (× 60 magnification). **e** Western blot analysis of FOXC1 and IGF-1R expression in HPAC cells treated with scramble siRNA (Scr) or IGF-1R siRNA or IGF-1R siRNA plus 100 nM of IGF-1 or 100 nM of IGF-1 alone. **f** MTS assay was used to measure the cell proliferation in response to IGF-1 treatment (5, 50, 100, and 200 nM) in parental and FOXC1-silenced HPAC cells. **g** Western blot analysis of IGF-1R, FOXC1, pAKT, Cyclin D1, and Vimentin in HPAC cells treated with FOXC1 siRNA or IGF-1 or FOXC1 siRNA plus IGF-1. **h** Western blot analysis of FOXC1 and IGF-1R expression in parental and FOXC1-overexpressing MIA PaCa-2 cells treated with or without Picropodophyllin (PPP). **i** Gaussia luciferase assay was used to show FOXC1 promoter activity in MIA PaCa-2 cells in the presence of IGF-1 or PPP. Data shown as mean ± SEM. Experiments (*n* = 3) were repeated three times in triplicates. *p < 0.05

Interestingly, altering the expression of FOXC1 directly impacted the expression levels of IGF-1R, suggesting a possible crosstalk between IGF-1R and FOXC1 (Fig. 2a–d). Furthermore, immunofluorescence analysis demonstrated that exogenous overexpression of FOXC1 in MIA PaCa-2 cells increased the expression of IGF-1R in both the cell membrane and nucleus, while the control cells expressed IGF-1R only on the cell membrane (Fig. 2d). Treatment of IGF-1 to IGF-1R-silenced HPAC cells was not effective in increasing cell proliferation, which demonstrates that IGF-1-induced cell proliferation is facilitated by IGF-1R (Supplementary Fig. S2). In addition, IGF-1 treatment to IGF-1R-silenced HPAC cells did not rescue the FOXC1 expression level compared with the scrambled control and IGF-1-treated parental HPAC cells (Fig. 2e). These data indicate the significance of the crosstalk between IGF-1R and FOXC1 in pancreatic cancer. To further understand the crosstalk between IGF-1R and FOXC1, we studied the cell proliferation in parental and FOXC1-silenced HPAC cells treated with IGF-1. IGF-1 treatment in parental HPAC cells increased cell proliferation in a dose-dependent manner, while there was no significant effect in IGF-1 treatment to FOXC1-silenced HPAC cells, which demonstrates that IGF-1-induced cell proliferation is facilitated by FOXC1 (Fig. 2f). To further validate the role of FOXC1 in IGF-1/IGF-1R signaling, parental HPAC cells and FOXC1-silenced HPAC cells were treated with IGF-1 and its effect on key marker of cell proliferation, cell cycle, and EMT was analyzed. Silencing FOXC1 reduced the expression levels of vital markers involved in proliferation (pAKT), cell cycle (Cyclin D1), and EMT (Vimentin). IGF-1 treatment to parental HPAC cells increased expression of pAKT, Cyclin D1, and Vimentin. On contrary, IGF-1 treatment to FOXC1-silenced HPAC cells was not able to significantly rescue the expression of any of the markers, which were decreased in response to FOXC1 silencing (Fig. 2g). These data further demonstrate that FOXC1 mediates IGF-1/IGF-1R signaling in several cellular processes involved in pancreatic cancer growth and progression.

In another set of experiments, IGF-1R was inhibited using picropodophyllin (PPP) and its effect on cell proliferation was measured. To begin with, a PPP dose-response study (0.1, 0.5, 1.0, 1.5, 2.0 µM of PPP) was done using MIA PaCa-2 cells. Cell proliferation decreased with increasing doses of PPP (Supplementary Fig. S3A). To confirm that the dose-dependent decrease in cell proliferation was due to PPP-induced inhibition of IGF-1R, we analyzed the levels of IGF-1R protein in response to the various doses of PPP. Our data clearly demonstrated that with increasing doses of PPP there was a corresponding decrease in the levels of IGF-1R (Supplementary Fig. S3B). Based on these data, 2 µM PPP concentration was selected for further studies. To further elucidate whether IGF-1R regulated cell proliferation through FOXC1, we inhibited IGF-1R using PPP in FOXC1-overexpressing MIA PaCa-2 cells. Inhibition of IGF-1R in FOXC1-overexpressing MIA PaCa-2 cells was not very effective in decreasing cell proliferation (Supplementary Fig. S3C). We also observed that decreased expression of IGF-1R in response to PPP had also resulted in remarkably reduced the expression of FOXC1 in parental HPAC cells. On the other hand, overexpression of FOXC1 maintained the expression levels of IGF-1R even in the presence of PPP (Fig. 2h), indicating the positive feedback regulation between IGF-1R and FOXC1, which enhances pancreatic cancer growth. Further, to determine the regulation of FOXC1 by IGF-1/IGF-1R, we performed FOXC1 promoter reporter analysis. Gaussia luciferase (GLuc) reporter assay revealed that IGF-1 increased FOXC1 promoter activity, while PPP treatment significantly reduced FOXC1 promoter activity (Fig. 2i). Taken together, all these data suggest that IGF-1R and FOXC1 regulate each other and FOXC1 is a direct downstream signaling molecule of IGF-1/IGF-1R axis.

### FOXC1 influences cell proliferation and survival through P13K/AKT/mTOR pathway in pancreatic cancers

**Fig. 3:**
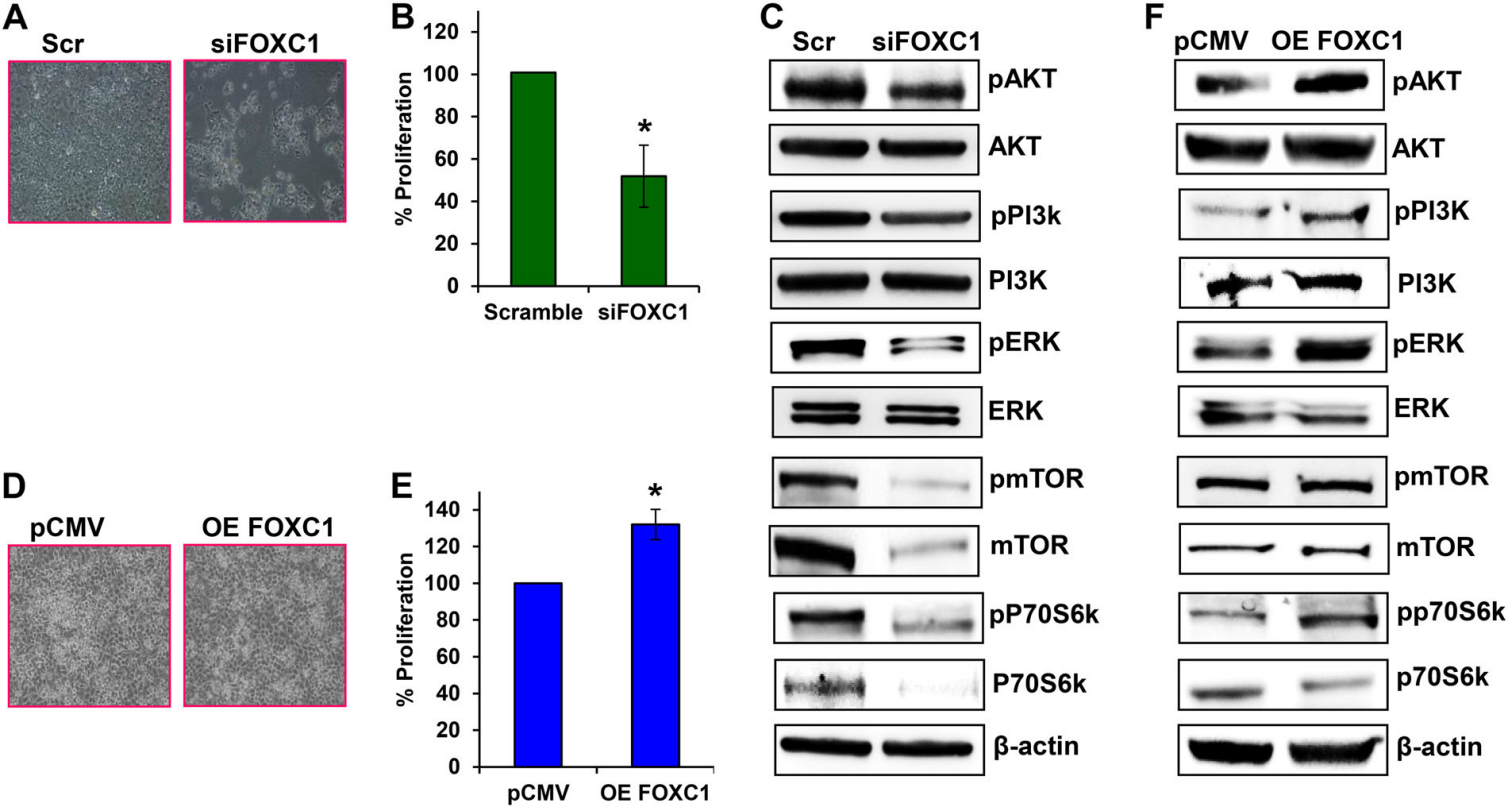
Effect of FOXC1 on pancreatic cancer cell proliferation. **a** Morphological changes occurred in FOXC1-silenced HPAC cells, which were observed under a light microscope at × 10 magnification. **b** MTS assay was used to measure cell proliferation of FOXC1-silenced HPAC cells. **c** Western blot analysis of proliferation markers in FOXC1-silenced HPAC cells. **d** Morphological changes occurred in FOXC1-overexpressing MIA PaCa-2 cells, which were observed under a light microscope at × 10 magnification. **e** MTS assay was used to measure the proliferation of FOXC1-overexpressing MIA PaCa-2 cells. **f** Western blot analysis of proliferation markers in FOXC1-overexpressing MIA PaCa-2 cells. Results represented as mean ± SEM. Data shown as mean ± SEM. Experiments (*n* = 3) were repeated three times in triplicates. *p < 0.05

Silencing FOXC1 exhibited significant morphological changes and increased cell death in HPAC cells (Fig. 3a). Further, cell proliferation was also significantly decreased in the FOXC1-silenced HPAC cells (~50%) (Fig. 3b). It was found that silencing FOXC1 in HPAC cells significantly decreased expression levels of the active forms of AKT, phospatidyl inositol 3-kinase (PI3K), and extracellular signal-regulated kinase (ERK), as well as both the active and total forms of mammalian target of rapamycin (mTOR) and p70s6k (Fig. 3c). Immunofluorescence imaging also verified the reduced expression of proliferative marker Ki67 in response to suppression of FOXC1 (Supplementary Fig. S4). Overexpression of FOXC1 increased the cell proliferation of MIA PaCa-2 cells by 132% (Fig. 3d, e). In addition, overexpression of FOXC1 in MIA PaCa-2 cells resulted in increasing the active forms of AKT, PI3K, ERK, and p70s6k (Fig. 3f). However, overexpression of FOXC did not alter the expression of both active and total forms of mTOR; interestingly, the expression of total form of p70s6k was decreased. These data reveal that FOXC1 alters the proliferative and survival potential of pancreatic cancer cells through the PI3K/AKT/mTOR signaling pathway.

### FOXC1 regulates migration, invasion, and anchorage-independent growth of pancreatic cancer cells

**Fig. 4:**
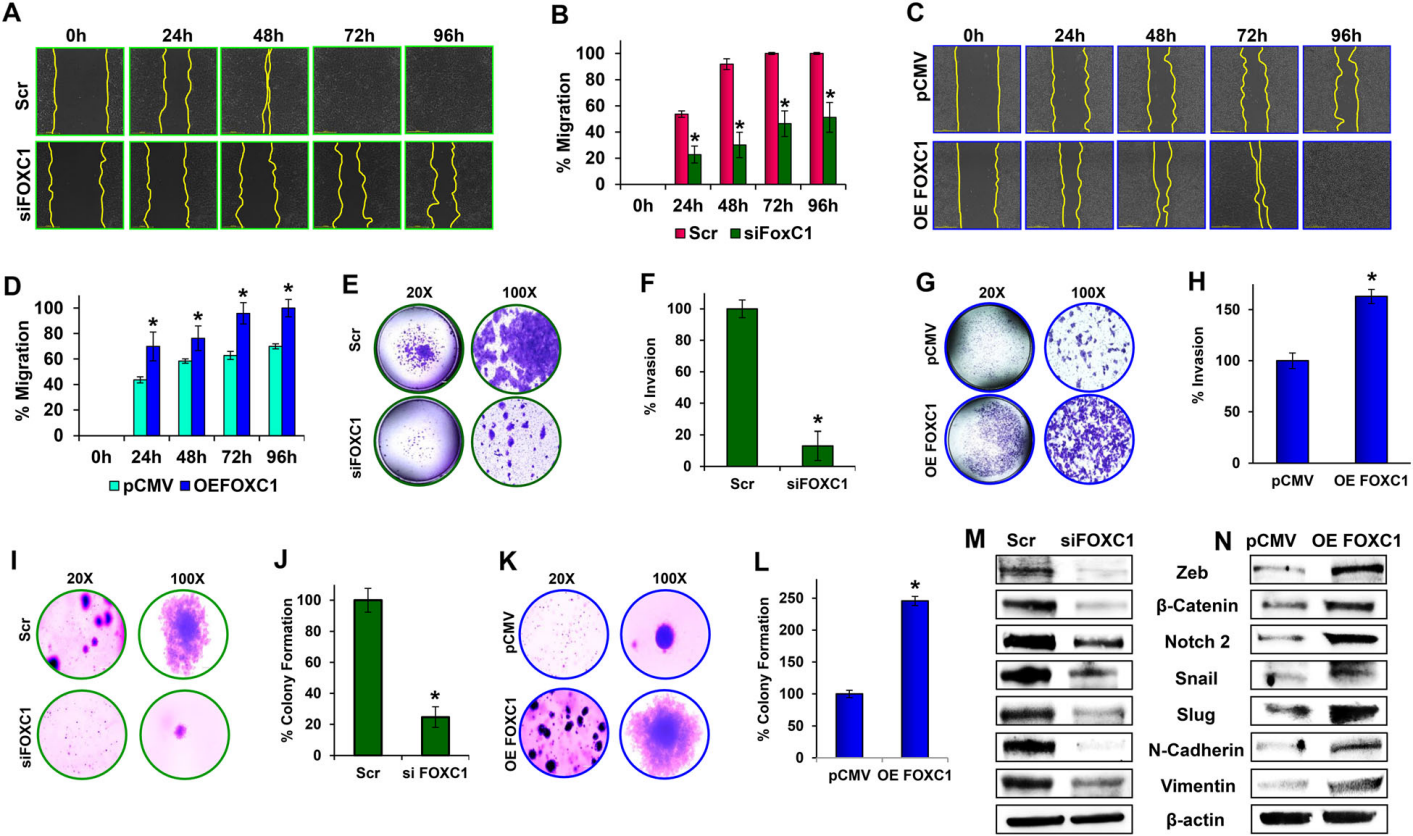
Effect of FOXC1 on metastasis of pancreatic cancer cells. **a** Wound-healing assay was performed in control (Scr) and FOXC1-silenced (siFOXC1) HPAC cells; migration was analyzed using an automated Nikon Biostation CT at 2 h intervals for up to 96 h at × 4 magnification. **b** Quantitative analysis of migration in FOXC1-silenced HPAC cells was calculated using NIS-Element AR software. **c** Wound-healing assay was performed in control (empty vector) and FOXC1-overexpressing (OE FOXC1) MIA PaCa-2 cells; migration was analyzed using an automated Nikon Biostation CT at 2 h intervals for up to 96 h at × 4 magnification. **d** Quantitative analysis of migration in FOXC1-overexpressing MIA PaCa-2 was calculated using NIS-Element AR software. **e** Invasiveness of FOXC1-silenced HPAC cells was observed using a Matrigel invasion assay (left: × 20 magnification; right: × 100 magnification). **f** Percentage invasion of control (Scr) and FOXC1-silenced (siFOXC1) HPAC cells was obtained by counting invaded cells from each group. **g** Invasiveness of FOXC1-overexpressing MIA PaCa-2 cells was observed using a Matrigel invasion assay (left: × 20 magnification; right: × 100 magnification). **h** Percentage invasion of control (pCMV) and FOXC1-overexpressing (OE FOXC1) MIA PaCa-2 cells was obtained by counting invaded cells from each group. **i** Colony formation assay was performed with control (Scr) and FOXC1-silenced (siFOXC1) HPAC cells (left: × 20 magnification; right: × 100 magnification). **j** The percentage of colonies were calculated with parental HPAC control cells serving as the baseline. **k** Colony formation assay was performed with control (pCMV) and FOXC1-overexpressing (OE FOXC1) MIA PaCa-2 cells (left: × 20 magnification; right: × 100 magnification). **l** The percentage of colonies were calculated with parental MIA PaCa-2 control cells serving as the baseline. **m** Western blot analysis of EMT markers in FOXC1-silenced HPAC cells and **n** FOXC1-overexpressing MIA PaCa-2 cells. Data shown as mean ± SEM. Experiments (*n* = 3) were repeated three times in triplicates. *p < 0.05

Silencing FOXC1 clearly inhibited the migratory abilities of HPAC cells. By 72 h, the parental HPAC cells migrated completely, closing the wound while even at 96 h, the FOXC1-silenced cells had only migrated about 50%. (Fig. 4a, b). To further verify the role of FOXC1 on cancer cell migration, a scratch assay was performed using FOXC1-overexpressing MIA PaCa-2 cells. The parental MIA PaCa-2 cells are not highly aggressive and did not migrate completely by 96 h, whereas FOXC1-overexpressing cells migrated within 96 h, demonstrating the pro-migratory effect of FOXC1 (Fig. 4c, d).

Matrigel-coated Boyden chambers were used to evaluate and examine the impact of FOXC1 on invasive capabilities of pancreatic cancer cells. Silencing FOXC1 drastically reduced the invasive capabilities by ~87% compared with the scramble control (Fig. 4e, f). On the other hand, when FOXC1 was overexpressed, there was an increase in invasive capabilities by ~63% compared with empty vector control (Fig. 4g, h).

Next, soft agar colony formation assay was performed to examine the impact of FOXC1 on cellular-anchorage-independent growth. Silencing FOXC1 greatly inhibited the colony-forming capabilities of HPAC cells (Fig. 4i). Quantitative analysis showed that colony formation was decreased by ~75% in FOXC1-silenced cells (Fig. 4j). In addition, FOXC1 overexpression increased the size and number of colonies formed by ~150% (Fig. 4k, l). Migration, invasion, and colony formation assay data clearly demonstrate that FOXC1 is an oncogenic TF, which could be a potential therapeutic target to inhibit metastatic capabilities of pancreatic cancers.

### FOXC1 regulates EMT transition

EMT is a hallmark of metastatic progression^22^. FOXC1-silenced HPAC cells were tested for key EMT markers such as Zeb, β-Catenin, Notch-2, Snail, Slug, N-Cadherin, and Vimentin. Suppression of FOXC1 drastically reduced the expression of all above-mentioned EMT markers compared with the scrambled control (Fig. 4m). Immunofluorescence imaging also verified the reduced expression of mesenchymal marker N-Cadherin in response to suppression of FOXC1 (Supplementary Fig. S5). On the other hand, when FOXC1 was overexpressed in MIA PaCa-2 cells, EMT markers were upregulated compared with the empty vector control group (Fig. 4n). This further suggests that FOXC1 has a critical role in regulating the EMT process.

### Suppression of FOXC1 inhibits key markers that promote angiogenesis

Previous research has demonstrated that FOXC1 has an active role in angiogenesis and specifically interacts in the VEGFR2-DLL4-Notch signaling pathway^23^. Our results suggested that silencing FOXC1 diminished the expression levels of angiogenesis markers, VEGFR2, and delta-like ligand protein 4 (DLL4) (Supplementary Fig. S6A). Immunofluorescence imaging also verified the reduced expression of VEGFR2 in response to FOXC1 suppression (Supplementary Fig. S6B). These results indicate that FOXC1 may enhance the aggressiveness of pancreatic cancer by increasing these key makers for angiogenesis.

### Effect of FOXC1 on tumor growth and EMT markers in pancreatic cancer xenograft model

**Fig. 5:**
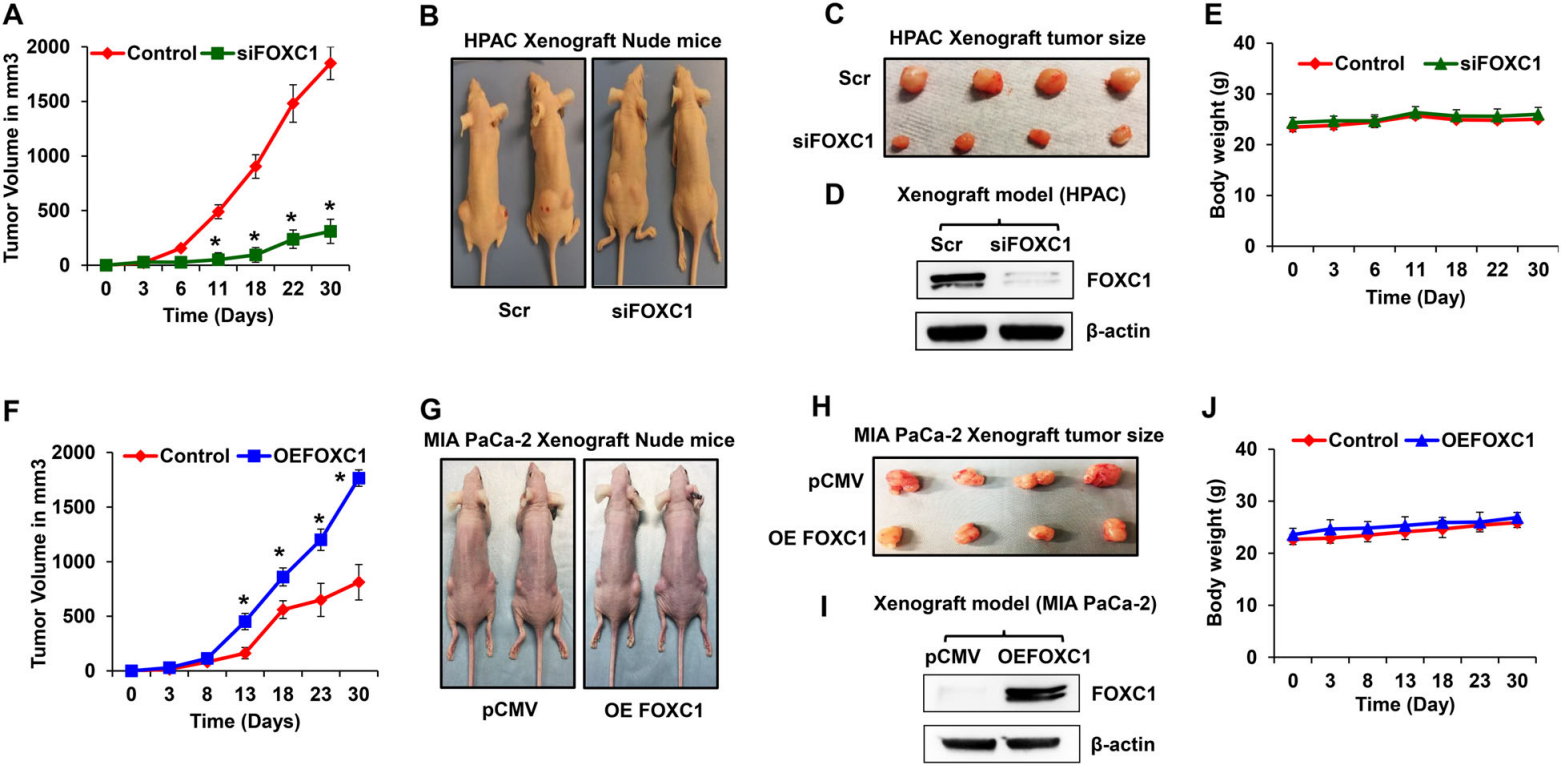
Effect of FOXC1 on in vivo tumor growth. **a** The tumor growth curve of FOXC1-silenced HPAC xenografts. **b** Representative photographs of mice subcutaneously injected with parental or siFOXC1-silenced HPAC cells. **c** Representative photographs of HPAC xenograft tumors excised from the nude mice. **d** Western blot analysis of FOXC1 protein expression in HPAC xenograft tumors. **e** Body weight of HPAC xenografted nude mice. **f** The tumor growth curve of FOXC1-overexpressing MIA PaCa-2 xenografts. **g** Representative photographs of mice subcutaneously injected with parental or FOXC1-overexpressing MIA PaCa-2 cells. **h** Representative photographs of MIA PaCa-2 xenograft tumors excised from the nude mice. **i** Western blot analysis of FOXC1 protein expression in MIA PaCa-2 xenograft. **j** Body weight of MIA PaCa-2-xenografted nude mice. Data shown as mean ± SEM. Experiments (*n* = 6 mice/group) were repeated three times. *p < 0.05

To validate our in vitro findings, we performed xenograft studies using FOXC1-silenced HPAC and FOXC1-overexpressing MIA PaCa-2 cells. Different sets of athymic nude mice (*n* = 6) received subcutaneous implantations of FOXC1-silenced HPAC cells or FOXC1-overexpressing MIA PaCa-2 cells or their respective parental cells. FOXC1 silencing resulted in significant reduction of the tumor growth of HPAC xenografts (Fig. 5a–c). Immunoblot data confirmed the reduced expression of FOXC1 in xenograft tumor tissues (Fig. 5d). Animals with FOXC1-silenced HPAC xenografts did not have any change in the body weight compared with controls (Fig. 5e). Meanwhile, overexpression of FOXC1 accelerated the tumor growth of MIA PaCa-2 xenografts (Fig. 5f–h). The xenograft tumors had increased expression of FOXC1 (Fig. 5i). No change in the body weight was observed in FOXC1-overexpressing MIA PaCa-2-xenografted mice (Fig. 5j). Both silencing and overexpression of FOXC1 exhibited the vital role of FOXC1 in pancreatic tumor growth.

**Fig. 6:**
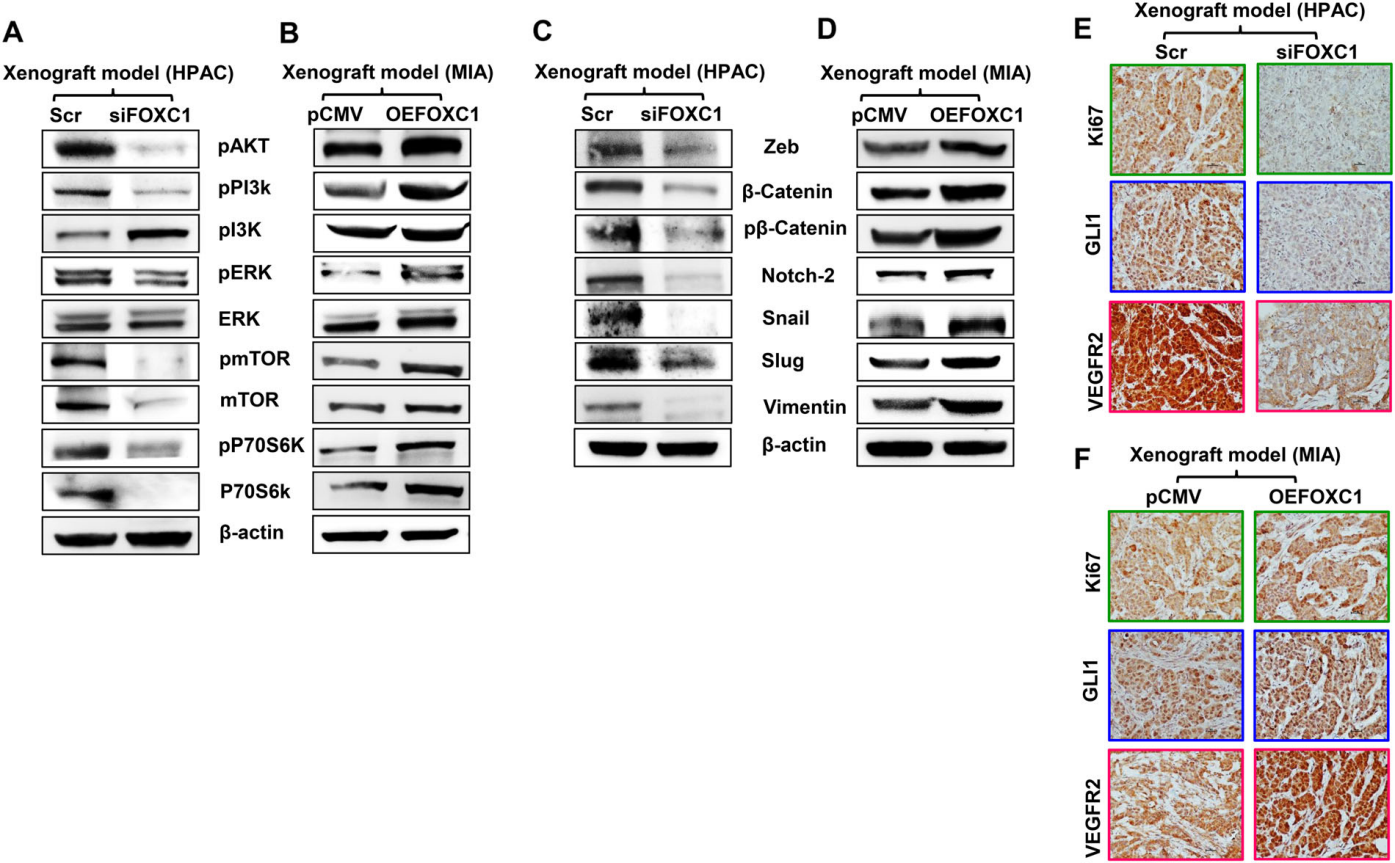
Effect of FOXC1 on proliferation and EMT of xenograft tumors. **a** Western blot analysis of proliferation markers in FOXC1-silenced HPAC xenograft tumors and **b** FOXC1-overexpressing MIA PaCa-2 xenograft tumors. **c** Western blot analysis of EMT markers in FOXC1-silenced HPAC xenograft tumors and **d** FOXC1-overexpressing MIA PaCa-2 xenograft tumors. **e** Immunohistochemical analysis of Ki67, Gli1, and VEGFR2 in FOXC1-silenced HPAC xenograft tumors and **f** FOXC1-overexpressing MIA PaCa-2 xenograft tumors. Data shown as mean ± SEM. Experiments (*n* = 3) were repeated three times. *p < 0.05

Similar to what we observed in our in vitro experiments, even in the in vivo experiments, suppression of FOXC1 resulted in significant downregulation of phosphorylated forms of PI3K, AKT, mTOR, ERK, and P70S6K (Fig. 6a). On the other hand, overexpression of FOXC1 increased these markers (Fig. 6b). Further, the expression levels of mesenchymal markers, such as zeb, β-catenin, pβ-catenin, notch-2, snail, slug, and vimentin were suppressed in the FOXC1-silenced group (Fig. 6c). As expected, these key markers of EMT were highly increased when FOXC1 was overexpressed (Fig. 6d). We examined the expression levels of proliferative marker Ki67, metastasis marker Gli1, and VEGFR2 in tumor tissues derived from the xenografts. IHC analysis confirmed the reduced levels of Ki67, Gli1, and VEGFR2 in FOXC1-silenced xenograft tumors (Fig. 6e), whereas FOXC1 overexpression upregulated these proliferative and metastatic markers compared with the xenograft tumors from empty vector control tumor group (Fig. 6f). These findings validate our in vitro observations and demonstrates the pro-tumorigenic role of FOXC1 in pancreatic cancer. Overall, these results indicate that FOXC1 suppression inhibits pancreatic cancer growth and progression.

## Discussion

The previous study from our lab demonstrated that IGF-1R was upregulated in pancreatic cancer and contributes to its progression and metastasis^15^. Growth factors regulate several TFs and it is well-known that TFs are altered/dysregulated during the pathogenesis of cancer. Dysregulation of TFs has been one of the contributing factors that play a role in the metastasis and tumorigenesis of cancers^24–26^. Targeting TFs as a potential therapeutic might provide promising treatments for pancreatic cancer^27^. Our results in the present study demonstrate that FOXC1 to be the most downregulated TF in response to IGF-1R silencing. Furthermore, the expression of FOXC1 and IGF-1R are interdependent, suggesting a strong positive feedback regulation between IGF-1R and FOXC1. Genetic alterations of IGF-1R can lead to FOXC1 overexpression, which has been associated with poor prognosis in several cancer types^16^. Earlier studies have shown that inhibition of IGF-1R activity reduced tumor growth and metastasis^28^ but had several adverse side effects. Hence, the present study focused to identifying the role of FOXC1 in pancreatic cancers growth and metastasis.

Earlier it has been reported that FOXC1 has an oncogenic role in breast, colon, liver, lung, and prostate cancer^18,29^. Our data demonstrate that FOXC1 mediates IGF-1R signaling by activating the transcription/translation of genes that are involved in crucial processes such as proliferation, cell survival, metastasis, and angiogenesis. Screening a panel of pancreatic cancer cell lines for FOXC1 expression revealed that aggressive pancreatic cancers express higher levels compared with less aggressive pancreatic cancer cell lines. In addition, immunohistochemical analysis of pancreatic adenocarcinomas also indicated that the expression of FOXC1 increases with advancing stages of pancreatic cancer.

Among the many pathways involved in cell proliferation, the PI3K/AKT and ERK signaling is one of the commonly dysregulated pathways in human cancers. In addition to cell proliferation, it is also involved in growth and metastasis^30^. In cervical cancers and melanoma, it was shown that FOXC1 influences cell proliferation, migration, and invasion through PI3K/AKT signaling pathway^31,32^. FOXC1 silencing and overexpression studies revealed that it regulates the PI3K/AKT and ERK signaling, favoring pancreatic cancer cell growth and proliferation.

Migration, invasion, EMT, and colony formation are key components that influence metastasis. Silencing the expression of FOXC1 decreased the migratory and invasive capabilities, whereas FOXC1 overexpression drastically increased these capabilities. The significant reduction in the size and number of colonies formed in response to FOXC1 silencing demonstrates that FOXC1 effectively promotes metastasis of pancreatic cancers. Furthermore, altering the expression levels of FOXC1 alters the expression of PI3K/AKT/mTOR/p70S6K/ERK/Zeb/β-Catenin/Notch-2/Snail/Slug/N-Cadherin and Vimentin. Suppression of FOXC1 decreased the levels of angiogenic markers VEGFR2 and DLL4 in pancreatic cancer cells. Nuclear expression of N-cadherin has been associated with poor prognosis for cancers such as the nasopharyngeal carcinomas^33^. Nuclear localization of VEGFR2 has been shown to activate its own promoter that could be involved in amplifying the angiogenic response. Further, nuclear VEGFR2 is expected to interact with several nuclear proteins, including the Sp1, which is a TF that influences genes required for angiogenesis^34^. Based on the available literature we believe that the nuclear expression of both N-cadherin and VEGFR observed in our study could be due to the aggressive nature of pancreatic cancers. These results suggest that FOXC1 has oncogenic properties that favors metastasis of pancreatic cancers.

Overall, our in vitro and in vivo data demonstrates that FOXC1 has a significant role in pancreatic cancer by increasing cell proliferation, migration, invasiveness, and metastatic capabilities. As a downstream signaling molecule of IGF-1R, FOXC1 has been shown to have the same effect on cancer cells in terms of angiogenesis, proliferation, tumorigenesis, and metastasis. Furthermore, FOXC1 is not known to be actively expressed in normal cells but is highly expressed in pancreatic cancer cells, which is key for its potential to become a novel therapeutic target for pancreatic cancer. Due to this specificity, targeting FOXC1 is expected to have less adverse side effects as a targeted therapy for pancreatic cancer patients. Based on our findings, we propose that FOXC1 can be used as a novel and potential therapeutic target for pancreatic cancer.

## Materials and methods

### Cell lines and reagents

HPAC, Capan-2, Capan-1, AsPC-1, PANC-1, MIA PaCa-2, and BxPC-3 were acquired from the American Type Culture Collection (ATCC, Manassas, VA, USA). HPAC, PANC-1, BxPC-3, and ASPC-1 cell lines were cultured in RPMI-1640 media supplemented with 10% fetal bovine serum (FBS), 100 units/mL of penicillin, and 100 μg/mL of streptomycin. MIA PaCa-2 cell line was cultured in Dulbecco’s modified Eagle’s media supplemented with 10% FBS, 2.5% horse serum, 100 units/mL of penicillin, and 200 mM glutamine. Capan-1 cells were cultured in ATCC-formulated Iscove’s modified Dulbecco’s medium supplemented with 20% FBS. Capan-2 cells were cultured in ATCC-formulated McCoy’s 5a medium modified complemented with 10% FBS.

The primary antibodies acquired and used for the various experiments were as follows: IGF-1R (3027), Notch-2 (4530P), Snail (3879), N-cadherin (4061), Zeb (3396), Vimentin (5741), Slug (9585), β-Catenin (9562), pPI3K (4228), PI3K (4292), pmTOR (2974), mTOR (4517), pp70S6 kinase (9206), p70S6 kinase (9202), VEGFR2 (9698), DLL4 (2589), Gli1 (3538), and Cyclin D1 (2978) (Cell Signaling Technology, Boston, MA, USA); pAKT (sc-101629), AKT (sc-5298), pERK (sc-101760), and ERK (sc-94) (Santa Cruz Biotechnology, Santa Cruz, CA, USA); Ki67 (ab15580) (Abcam, Cambridge, MA, USA); FOXC1 (ABD71) (EMD Millipore, Darmstadt, Germany); and β-actin (Sigma-Aldrich, St. Louis, MO, USA). Respective anti-mouse and anti-rabbit secondary antibodies were obtained from Santa Cruz Biotechnology.

### Silencing of IGF-1R and FOXC1 in HPAC cell line

Silencing of IGF-1R was performed as described in our earlier study^15^. IGF-1R-silenced cells were used for TF activation profiling, cell proliferation, gene, and protein expression experiments. Further, FOXC1 expression was silenced using siRNA targeting FOXC1 (Origene, Rockville, MD, USA). Scrambled siRNA was used as controls. Transfection was performed using MIrus bio TransIT siQUEST transfection reagent (Mirus Bio, Madison, WI, USA). Immunoblot analysis was performed to confirm the silencing of FOXC1.

### Sequences for FOXC1 siRNA subtypes “A,” “B,” and “C”

SR301606A — rGrGrArUrUrGrCrUrGrCrArArArUrArArArUrArCrArCrUTT

SR301606B — rArCrArCrCrArGrCrGrArArCrArGrArArUrArUrCrCrCrUCC

SR301606C — rCrCrArGrArUrArArCrArCrGrUrArArGrUrUrUrCrUrUrCTT

### Overexpression of FOXC1 in MIA PaCa-2 cells

DH5α competent cells were transformed with 2 µg of plasmid DNA for human FOXC1 or PCMV-XL6 cloning vector alone (Origene). The transformed cultures were spread on a pre-warmed 4% agar plate containing 100 µg/mL ampicillin at 37 °C overnight. Single colonies were selected and inoculated in Luria-Bertani broth for 12 to 16 h treated with 25 µg/mL ampicillin. FOXC1 plasmid transfected *Escherichia coli* cells were collected, DNA was isolated, and purified using Invitrogen Pure Link HiPure Plasmid Filter Maxiprep Kit (Life Technologies, Grand Island, NY, USA). MIA PaCa-2 cells were seeded in six-well plates at a density of 2.5 × 10^5^ cells/well; 24 h after seeding, cells were transfected with different concentrations (1, 5, and 10 μg) of FOXC1 pDNA. Transfection was performed using MIrus bio TransIT 2020 transfection reagent (Mirus Bio). The overexpression of FOXC1 was confirmed using immunoblottings and immunofluorescence.

### TF activation profiling plate array II

HPAC cells were seeded in a six-well plate at a density of 2.5 × 10^5^ cells/well to investigate the activity of 96 TFs. According to manufacturer’s protocol, 10 µg of nuclear protein from IGF-1R-silenced HPAC cells was used to measure the activity of TFs using the TF Activation Profiling Plate Array II (Signosis, Santa Clara, CA, USA). The signals were detected using a microplate reader (CLARIOstar, BMG LABTECH, Cary, NC, USA).

### Pancreas adenocarcinoma tissue array

Pancreas adenocarcinoma tissue microarray (TMA) was purchased from US Biomax, Inc. (Rockville, MD). The TMA contained two core sections of each formalin-fixed paraffin embedded samples (*n* = 24) of pancreatic adenocarcinoma at different stages and along with normal pancreatic tissues, which were subjected to immunohistochemistry (IHC) to measure the FOXC1 expression levels.

### Immunohistochemical analysis

TMA and xenograft tumor sections (siFOXC1-silenced HPAC or FOXC1-overexpressing MIA PaCa-2 and their respective control groups) were subjected to IHC analysis as described in our earlier study^15^. FOXC1, IGF-1R, Gli1, VEGFR2, and Ki67 expression was analyzed. IHC staining was quantitated at five random fields per section with at least 200 cells/field.

### Cell proliferation assay

IGF-1R or FOXC1-silenced HPAC cells and FOXC1-overexpressing MIA PaCa-2 cells were seeded in 96-well plates at a density of 0.3 × 10^4^ cells/well and treated with IGF-1 (5, 50, 100, and 200 nM) or PPP (0.1, 0.5, 1.0, 1.5, 2.0 µM of PPP). Twenty-four hours post treatment, MTS (3-(4,5-dimethylthiazol-2-yl)-5-(3-carboxymethoxyphenyl)-2-(4-sulfophenyl)-2H-tetrazolium) assay was performed to measure the cell proliferation using a microplate reader (CLARIOstar, BMG LABTECH).

### Scratch assay

The scratch assay was performed as described earlier^15^, to measure the influence of FOXC1 on pancreatic cancer cell migration using FOXC1-silenced HPAC (siFOXC1) and FOXC1-overexpressed MIA PaCa-2 cells (OE FOXC1). Monitoring and image capture was performed at 2 h intervals for a period of 96 h at 37 °C using the Biostation CT (Nikon Instruments, Inc. Melville, NY, USA). The distance migrated by the cells was calculated using NIS-Element AR software^15^.

### Invasion assay

The matrigel invasion assay was executed as described earlier^15^, in order to observe the impact of FOXC1 on the invasive capacity of pancreatic cancer cells. Five arbitrarily selected fields were captured using Nikon Eclipse TS 100 microscope at × 20 and × 100 magnification.

### Colony formation assay

Colony formation assay was performed using FOXC1-silenced HPAC cells and FOXC1-overexpressing MIA PaCa-2 cells as described before^15^. Colonies were counted manually and images were acquired using Nikon SMZ 1500 microscope at × 40 magnification.

### Immunofluorescence analysis

siFOXC1 or siIGF-1R HPAC cells and OE FOXC1 MIA PaCa-2 cells were used for immunofluorescence analysis of FOXC1, IGF-1R, Ki67, N-Cadherin, and VEGFR2. Slides were examined using Nikon laser scanning confocal microscope (Nikon Eclipse Ti, Nikon, NY USA).

### Quantitative RT-PCR

Total RNA was isolated from HPAC, Capan-2, Capan-1, AsPC-1, PANC-1, MIA PaCa-2, and BxPC-3 cells using the Trizol reagent (Invitrogen, Carlsbad, CA, USA). The appropriate FOXC1 and RRN18S primers (QIAGEN, Germantown, MD, USA) were used for this assay. The analyses were based on the comparative C_t_ method (2^−ΔΔCt^).

### FOXC1 promoter reporter GLuc assay

DH5α-competent cells were transformed with 2 µg of plasmid DNA using a promoter reporter clone for human FOXC1 (GeneCopoeia, Rockville, MD, USA). Transfections were performed as mentioned earlier. The transformed cultures were spread on a pre-warmed 4% agar plate containing 50 µg/mL kanamycin (Life Technologies) and incubated at 37 °C overnight. Single colonies were selected and inoculated in 50 µg/ml kanamycin-treated Luria-Bertani broth for 12–16 h. FOXC1 promoter plasmid DNA-transfected *E. coli* cells were collected, isolated, and purified using the Pure Link HiPure Plasmid Filter Maxiprep Kit (Life Technologies). MIA PaCa-2 cells were transfected with 5 µg of FOXC1 promoter reporter plasmid DNA or control plasmid DNA for 48 h. Post transfection, growth media containing 1.2 µg/ml of puromycin was used to select the cells, which stably expressed FOXC1 promoter DNA. These cells stably overexpressing FOXC1 were treated with IGF-1R inhibitor, PPP (2 µM) and IGF-1 (100 nM) for 24 h. After 24 h of incubation, 10 µL of the culture medium was mixed with 100 µL of luminescent substrate (1 ×) for GLuc or secreted alkaline phosphatase (SEAP) in a 96-well white plate (Secrete-Pair Dual Luminescence assay kit, Genecopoeia). GLuc and SEAP activity was measured using a luminometer (CLARIOstar, BMG LABTECH). Signal normalization was achieved by using the ratio of luminescent intensities of GLuc over internal standard control SEAP.

### Immunoblot analysis

Proteins from cultured cells and xenograft tumor tissues were extracted and used for expression studies as described earlier^15^ Protein expression was imaged using enhanced chemiluminescence (GE Las-4000).

### Xenograft studies

The Texas Tech University Health Sciences Center Institutional Animal Care and Use Committee approved all the animal experiments performed in this study. Six week old athymic nude mice were purchased from Harlan Laboratories (Madison, WI, USA). FOXC1-silenced HPAC cells and FOXC1-overexpressing MIA PaCa-2 cells, and their respective parental cells, were implanted subcutaneously in both the flanks (1.5 × 10^6^ cells/flank) of each animal (*n* = 6). All animals were monitored twice a week, and both tumor volume and body weight were recorded. After 1 month of observations, the mice were killed and the tumors were surgically excised. A portion of the tissue was fixed in 10% formalin for histopathological analysis and IHC, while the rest of the tissue was snap frozen in liquid nitrogen and used for molecular analysis.

### Statistical analysis

The data are expressed as mean ± SEM. Unpaired Student’s *t*-test was used to analyze differences between the control and experimental groups, using GraphPad Prism 5 software, version 5.03 (GraphPad Prism Software, San Diego, CA, USA). *P*-values < 0.05 were considered to be statistically significant.

## Electronic supplementary material

Supplementary Information

Supplementary Figure S1

Supplementary Figure S2

Supplementary Figure S3

Supplementary Figure S4

Supplementary Figure S5

Supplementary Figure S6
