# Supplementary material for: FOXC1 plays a crucial role in the growth of pancreatic cancer
Source: Oncogenesis. 2018 Jul 6;7(7):52. doi: 10.1038/s41389-018-0061-7 (PMC6033944; doi:10.1038/s41389-018-0061-7)
Supplement: Supplementary file 1 — Supplementary Information [file 41389_2018_61_MOESM1_ESM.docx]

**Supplementary Information: Figure Legends**

**Supplementary** **Fig. S1:**

**mRNA expression of FOXC1 in pancreatic cancer cell lines.** Relative mRNA expression level of FOXC1 was assessed by real-time RT-PCR in a panel of pancreatic cancer cell lines (HPAC, Capan-2, Capan-1, AsPC-1, PANC-1, MIA PaCa-2 and BxPC-3). RRN18S was used as the internal control for gene expression analysis. Data shown as mean ± standard error of the mean. Experiments (n=3) were repeated three times in triplicates. * indicates p<0.05.

**Supplementary** **Fig. S2:**

**IGF-1 induced IGF-1R signaling is crucial in pancreatic cancer cell proliferation.** MTS assay was used to measure cell proliferation in parental and IGF-1R-silenced HPAC cells treated with IGF-1. Data shown as mean ± standard error of the mean. Experiments (n=3) were repeated three times in triplicates. * indicates p<0.05.

**Supplementary Fig. S3:**

**FOXC1 facilitates IGF-1R signaling in pancreatic cancer cells. (A)** MTS assay was used to measure cell proliferation in MIA PaCa-2 cells treated with various concentrations (2, 1.5, 1, 0.5, and 0.1 µM) of picropodophyllin (PPP) for 24 hours. **(B)** Western blot analysis of IGF-1R expression in MIA PaCa-2 cells treated with 2, 1.5, 1, 0.5, and 0.1 µM PPP. **(C)** MTS assay was used to measure cell proliferation in FOXC1-overexpressing MIA PaCa-2 cells in the presence and absence of IGF-1R inhibitor (PPP). Data shown as mean ± standard error of the mean. Experiments (n=3) were repeated three times in triplicates. * indicates p<0.05.

**Supplementary Fig. S4:**

**Effect of FOXC1 on cell proliferation in HPAC cells.** Immunofluorescence analysis of proliferative marker Ki67 expression in FOXC1-silenced HPAC cells. Scramble control and siFOXC1 cells were fixed and stained with anti-Ki67 antibody and visualized through fluorescence confocal microscopy at 60X magnification. Data shown as mean ± standard error of the mean. Experiments (n=3) were repeated three times in triplicates. * indicates p<0.05.

**Supplementary Fig. S5:**

**Effect of FOXC1 on EMT in HPAC cells.** Immunofluorescence analysis of EMT marker N-Cadherin expression in FOXC1-silenced HPAC cells. Data shown as mean ± standard error of the mean. Experiments (n=3) were repeated three times in triplicates. * indicates p<0.05.

**Supplementary Fig. S6:**

**Effect of FOXC1 on angiogenesis in HPAC cells. (A)** Western blot analysis of angiogenic markers VEGFR2 and DLL4 in FOXC1-silenced HPAC cells. **(B)** Immunofluorescence analysis of angiogenesis marker VEGFR2 expression in FOXC1-silenced HPAC cells. Data shown as mean ± standard error of the mean. Experiments (n=3) were repeated three times in triplicates. * indicates p<0.05.
