# Supplementary figures and images for: FOXC1 plays a crucial role in the growth of pancreatic cancer
Source: Oncogenesis. 2018 Jul 6;7(7):52. doi: 10.1038/s41389-018-0061-7 (PMC6033944; doi:10.1038/s41389-018-0061-7)

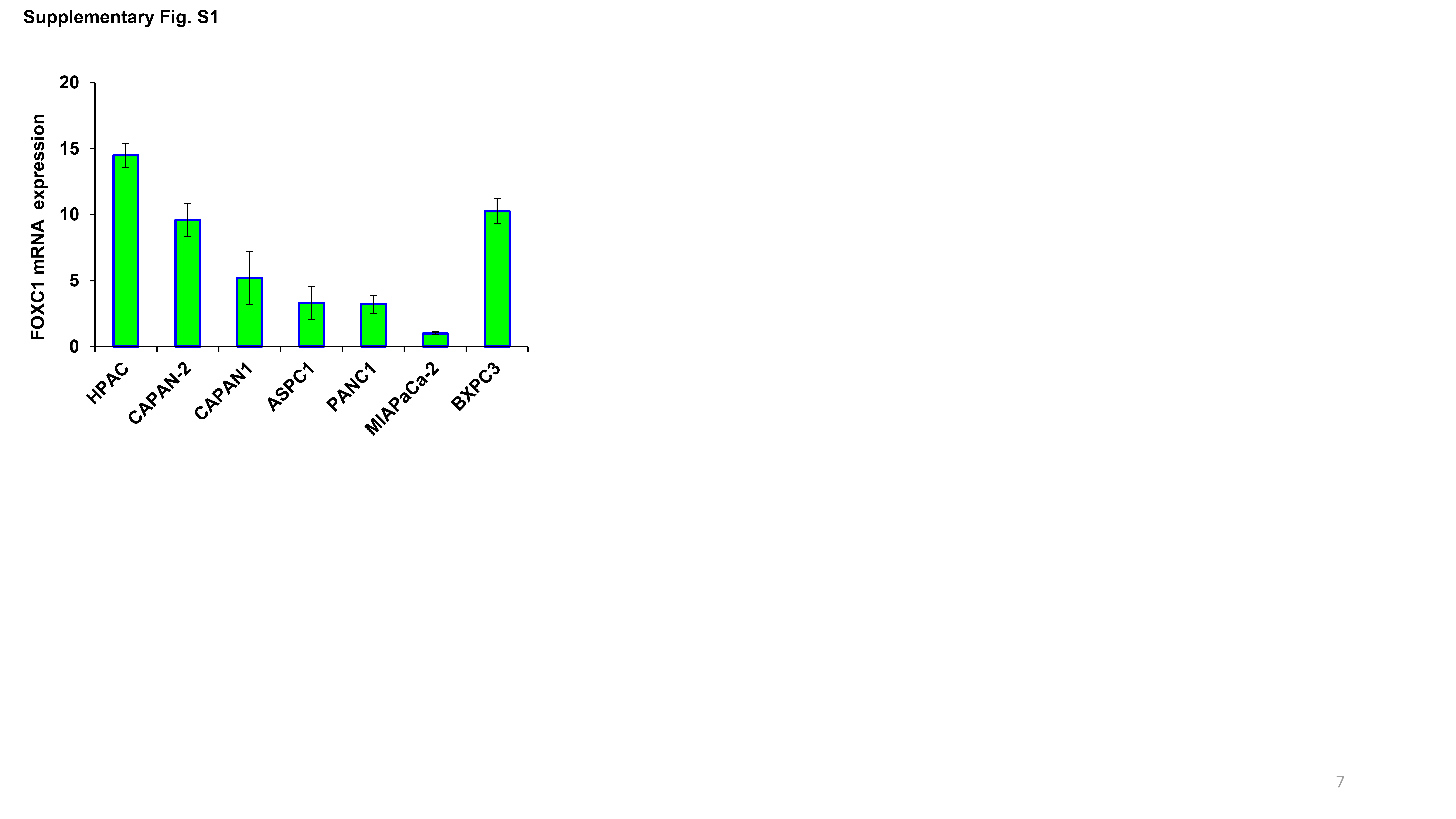

Supplement: Supplementary file 2 — Supplementary Figure S1 [file 41389_2018_61_MOESM2_ESM.tif]

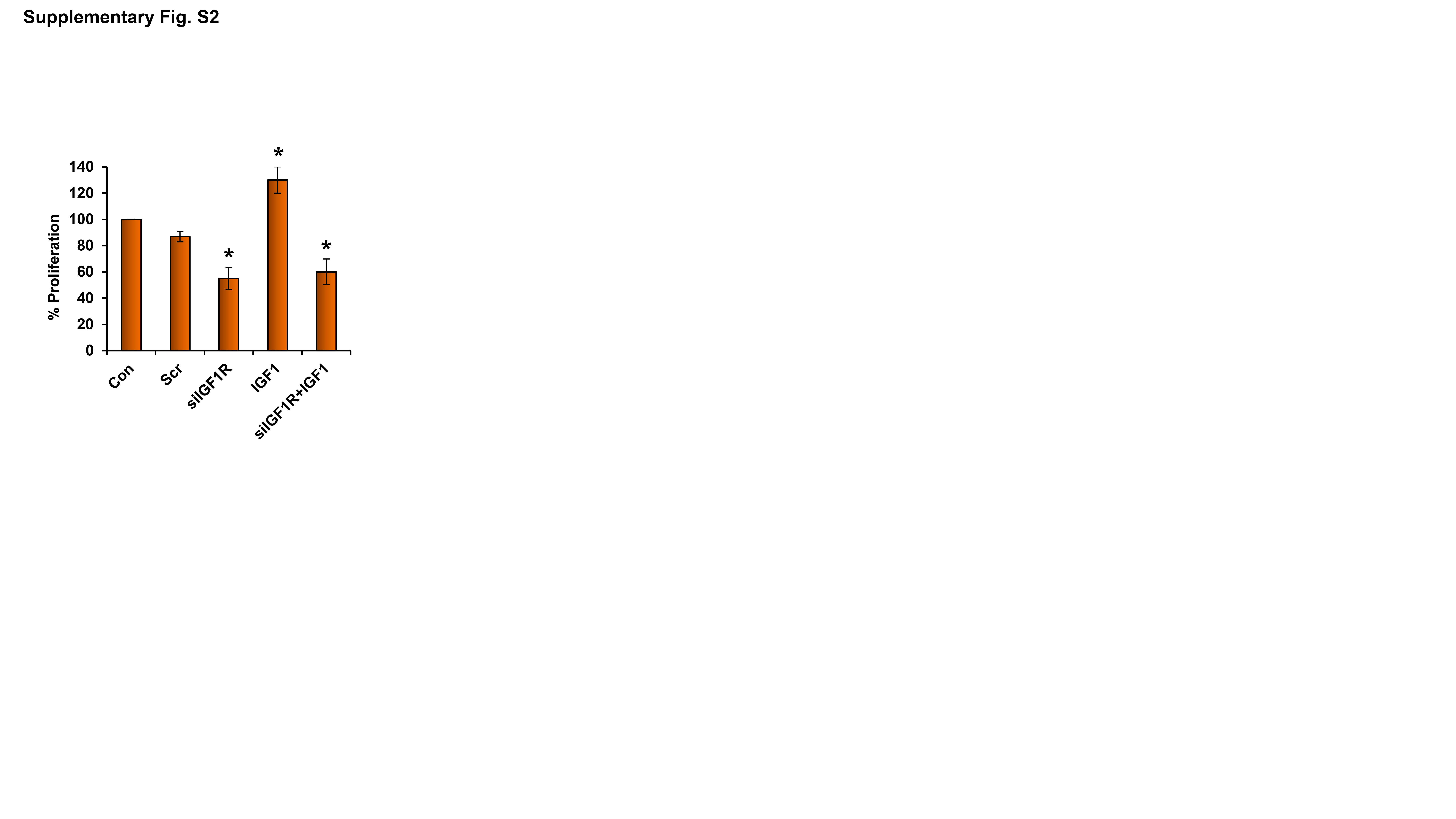

Supplement: Supplementary file 3 — Supplementary Figure S2 [file 41389_2018_61_MOESM3_ESM.tif]

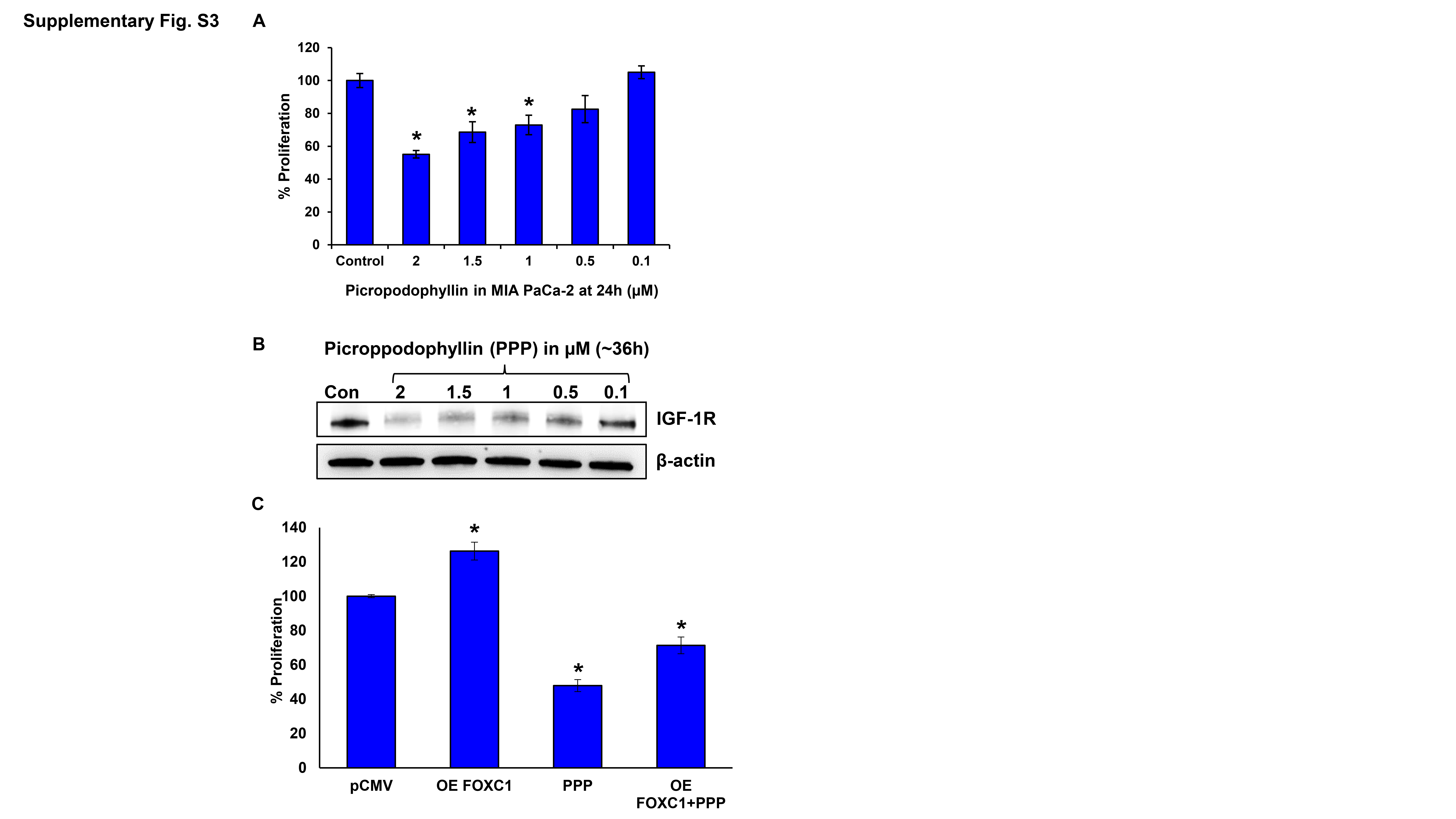

Supplement: Supplementary file 4 — Supplementary Figure S3 [file 41389_2018_61_MOESM4_ESM.tif]

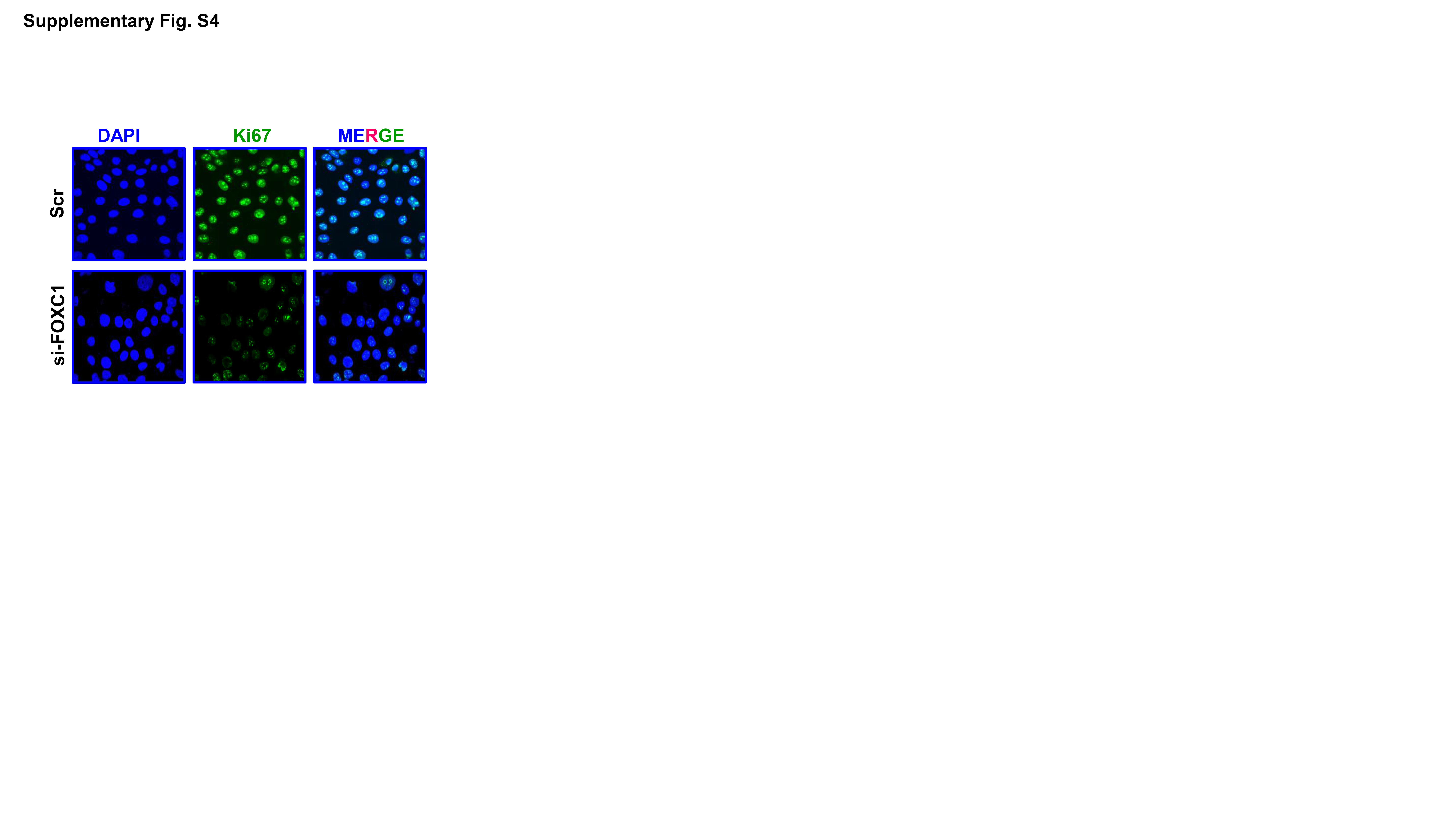

Supplement: Supplementary file 5 — Supplementary Figure S4 [file 41389_2018_61_MOESM5_ESM.tif]

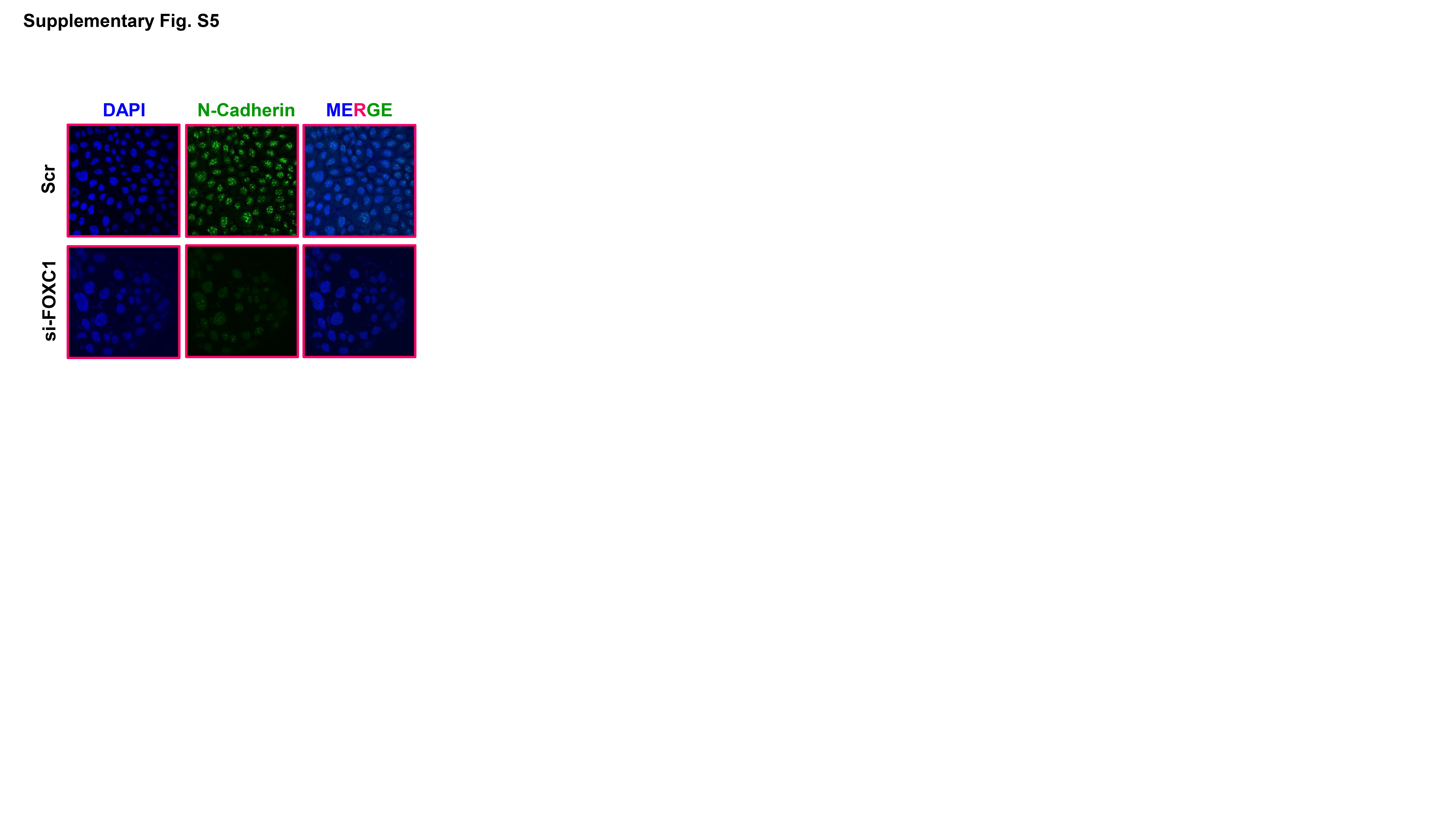

Supplement: Supplementary file 6 — Supplementary Figure S5 [file 41389_2018_61_MOESM6_ESM.tif]

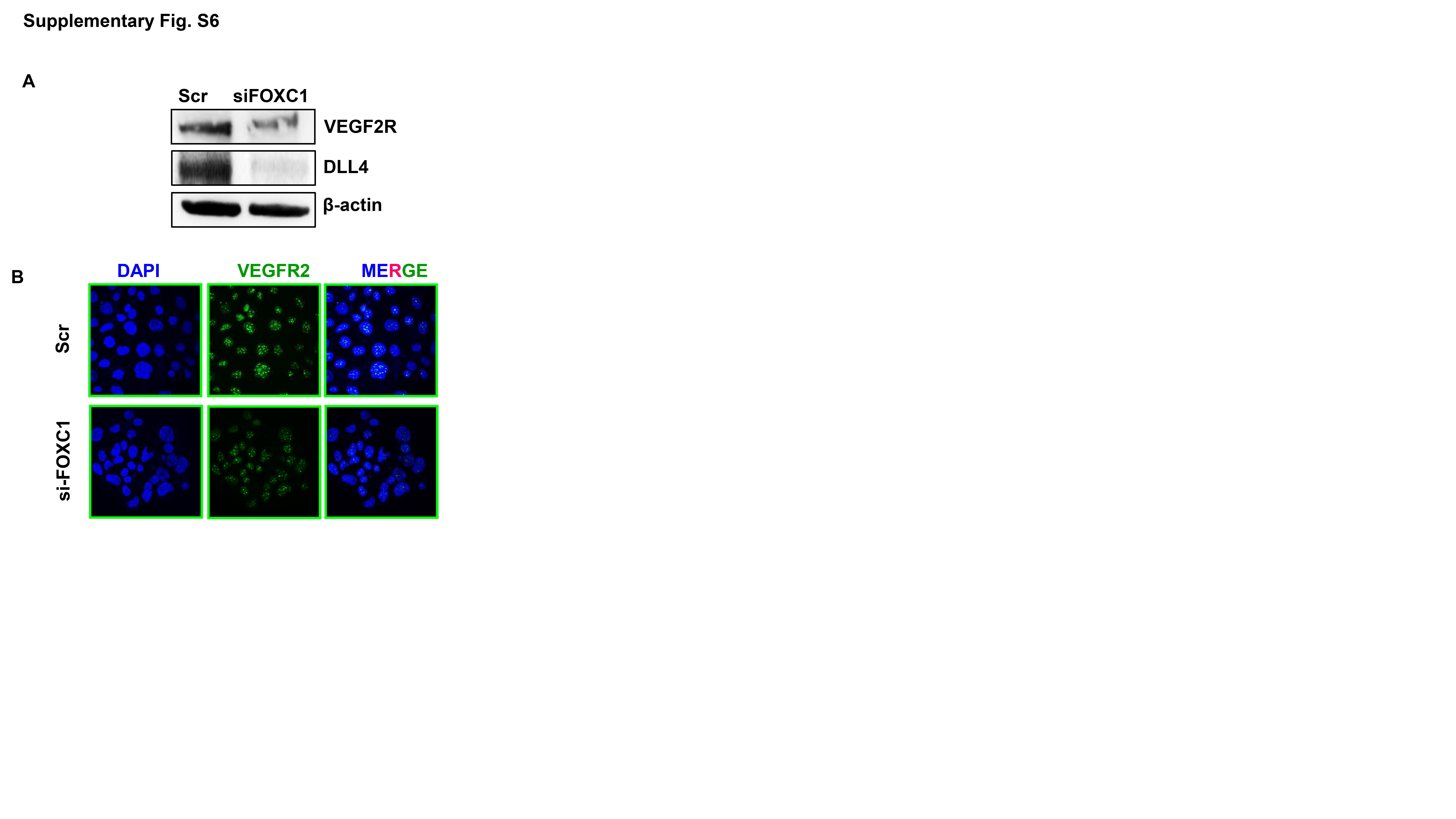

Supplement: Supplementary file 7 — Supplementary Figure S6 [file 41389_2018_61_MOESM7_ESM.tif]
